# Hyperventilation worsens inflammatory lung injury in spontaneously breathing rats

**DOI:** 10.36416/1806-3756/e20240269

**Published:** 2024-11-26

**Authors:** Juliana Dias Nascimento Ferreira, Maycon Moura Reboredo, Eduardo Leite Vieira Costa, Lídia Maria Carneiro da Fonseca, Jaime Retamal, Fabrício Júnio Mendes Santos, Flavia de Paoli, Adenilson de Souza da Fonseca, Leda Marília Fonseca Lucinda, Bruno Valle Pinheiro

**Affiliations:** 1. Divisão de Pneumologia e Terapia Intensiva, Hospital Universitário, Universidade Federal de Juiz de Fora, Juiz de Fora (MG), Brasil.; 2. Faculdade de Medicina, Universidade Federal de Juiz de Fora, Juiz de Fora (MG), Brasil.; 3. Centro de Biologia da Reprodução, Universidade Federal de Juiz de Fora, Juiz de Fora (MG), Brasil.; 4. Laboratório de Pneumologia, Laboratório de Investigação Médica 09, Disciplina de Pneumologia, Instituto do Coração, Hospital das Clínicas, Faculdade de Medicina, Universidade de São Paulo, São Paulo (SP), Brasil.; 5. Instituto de Ensino e Pesquisa, Hospital Sírio-Libanês, São Paulo (SP), Brasil.; 6. Departamento de Medicina Intensiva, Facultad de Medicina, Pontificia Universidad Católica de Chile, Santiago, Chile.; 7. Departamento de Morfologia, Instituto de Ciências Biológicas, Universidade Federal de Juiz de Fora, Juiz de Fora (MG), Brasil.; 8. Departamento de Biofísica e Biometria, Instituto de Biologia Roberto Alcantara Gomes, Universidade Estadual do Rio de Janeiro, Rio de Janeiro (RJ), Brasil.

**Keywords:** acute lung injury, acute respiratory distress syndrome, spontaneous breathing, hyperventilation, lung injury

## Abstract

**Objectives:**

Here, we investigated the effects of hyperventilation on acute lung injury (ALI) in spontaneously breathing rats.

**Methods:**

Wistar rats were randomized to receive either intraperitoneal lipopolysaccharides (LPS) or saline, and intravenous infusion of NH_4_Cl (to induce metabolic acidosis and hyperventilation) or saline. Four groups were established: control-control (C-C), control-hyperventilation (C-HV), LPS-control (LPS-C), and LPS-hyperventilation (LPS-HV). Venous blood gases were collected before and after NH_4_Cl infusion and analyzed to confirm the presence of metabolic acidosis and hyperventilation. After euthanasia, lung injury was assessed using the ALI score, morphometric quantification of perivascular edema, neutrophil counts in the bronchoalveolar lavage, and mRNA expression of biological markers in the lung tissue.

**Results:**

Hyperventilation induced inflammatory lung injury in previously healthy lungs and exacerbated injuries previously induced by LPS (ALI score: C-C=0.14 [IQR 0.12; 0.14]; C-HV=0.36 [IQR 0.31; 0.37]; LPS-C=0.51 [IQR 0.50; 0.54]; LPS-HV=0.58 [IQR 0.56; 0.62]; p<0.01). Perivascular edema, neutrophil counts in bronchoalveolar lavage, and amphiregulin mRNA expression were higher in the LPS-HV group compared to the control group.

**Conclusions:**

Hyperventilation increased inflammatory injury in rats with ALI during spontaneous ventilation. These results suggest that the impact of vigorous spontaneous breathing efforts on worsening inflammatory lung injury warrants further investigation.

## INTRODUCTION

Mechanical ventilation (MV) is an essential treatment for many patients with severe forms of acute lung injury (ALI).^1^ However, lung inflation during controlled positive pressure ventilation is heterogeneous, with greater expansion in the ventral regions compared to the dorsal regions. This ventilatory pattern worsens ventilation-perfusion (V/Q) matching and, consequently, impairs pulmonary gas exchange efficiency.^2^ Moreover, MV can cause uneven distribution of distending pressure, thus increasing the risk of ventilator-induced lung injury (VILI).^3^

Allowing spontaneous effort during MV can minimize the heterogeneous distribution of distending pressure, increase lung aeration, and improve V/Q matching and oxygenation.^4^ Additional potential benefits include enhanced hemodynamic performance, prevention of diaphragmatic atrophy, improved patient comfort, and a reduced need for aggressive sedation and/or paralysis.^5^

Conversely, recent evidence suggests that spontaneous efforts may contribute to lung injury, especially in severely injured lungs or when these efforts are vigorous. These efforts reduce pleural pressure (Ppl), increasing transpulmonary pressure and tidal volume (V_T_), both of which are associated with lung injury.^6^ ^,^ ^7^ In severely injured lungs with heterogeneous aeration, spontaneous efforts can also heighten local lung stress, leading to lung injury even with low V_T_.^8^ Additionally, a negative Ppl increases transmural vascular pressure (the difference between intravascular and extravascular pressure), promoting fluid leakage into the pulmonary interstitium and alveolar space.^9^ This effort-related lung injury is termed patient self-inflicted lung injury (P-SILI).^10^

During the COVID-19 pandemic, the concept of P-SILI was extended to patients not receiving mechanical ventilatory support. Some authors have postulated that strong spontaneous inspiratory efforts may exacerbate lung injury and edema by increasing tissue stress and pulmonary transvascular pressure. They advocated for early intubation and effective sedation, with or without paralysis, to break this cycle.^11^ However, the extent to which inspiratory efforts are capable of aggravating lung injury, and whether the potential benefits of early intubation outweigh the risks associated with invasive MV, remains unclear. Due to the lack of robust evidence, some authors have questioned early intubation, favoring noninvasive strategies for patients with acute hypoxemic respiratory failure, suggesting that the liberal use of intubation and MV may increase mortality in these patients.^12^

In the present study, we used an established model of ALI induced by lipopolysaccharides (LPS) to investigate whether hyperventilation caused by metabolic acidosis exacerbates lung injury in rats during spontaneous ventilation.

## METHODS

Adult male Wistar rats, weighing 280-360 g, were obtained from the Reproduction Biology Center of the Federal University of Juiz de Fora vivarium, in Minas Gerais, Brazil. This choice was made due to their favorable biological and physiological similarity to humans.^13^ The number of animals used followed the reduction principle, with an anticipated range of 6-8 animals per experimental group, consistent with previous studies conducted by our research team.^14^ ^,^ ^15^ The study was approved by the Animal Research Ethics Committee of the Federal University of Juiz de Fora (Protocol No.: 017/2022).

### Experimental protocol

Initially, the rats were randomly assigned to two main groups: the LPS group and the control group. In the LPS group, the animals received 5 mg/kg LPS (serotype 055:B5; Sigma-Aldrich, Israel) intraperitoneally, dissolved in 0.5 mL of 0.9% saline solution. Meanwhile, the animals from the control group received an equivalent volume of 0.9% saline solution intraperitoneally.

After 24 hours, the rats were anesthetized with an intraperitoneal bolus of xylazine (8 mg/kg), midazolam (5 mg/kg), and ketamine (80 mg/kg). A polyethylene catheter was inserted into the left jugular vein for infusion of the solutions and to obtain blood samples for venous blood gas analysis (ABL90 FLEX; Radiometer, Copenhagen, Denmark).

**Figure 1:**
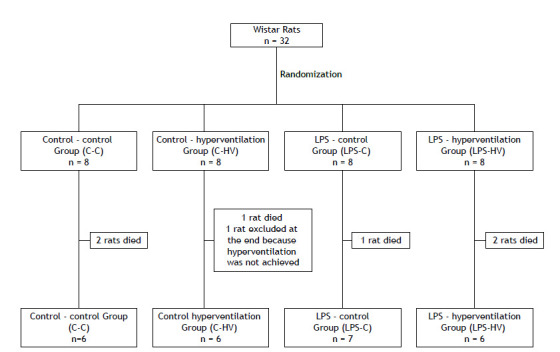
Animals included in the analyses.

The rats in the LPS and control groups were subsequently randomized into two subgroups, hyperventilation and control, resulting in four groups: control-control (C-C), control-hyperventilation (C-HV), LPS-control (LPS-C), and LPS-hyperventilation (LPS-HV) (Figure 1). The animals assigned to the hyperventilation groups (C-HV and LPS-HV) received intravenous infusions of 1.0 mL/kg of ammonium chloride (5 M-NH_4_Cl; Pharmacology Laboratory, School of Pharmacy, Federal University of Juiz de Fora, Brazil) dissolved in 0.9% saline solution, totaling 3.5 mL over 180 minutes. The animals in the control groups (C-C and LPS-C) received venous infusions of the same volume of 0.9% saline solution over 180 minutes. In order to assess the success of inducing metabolic acidosis (HCO_3_^-^ ≤ 15 mEq/L) and hyperventilation (reduction in PvCO_2_ ≥ 25% compared to baseline), a venous blood sample was collected for gas analysis immediately after the infusion ended.

After infusion with NH_4_Cl or saline, a tracheostomy was performed, and the trachea was cannulated with a 14-gauge tube. The rats were mechanically ventilated (Inspira ASV, Harvard Apparatus, USA) for one minute under the following parameters: volume-controlled mode, V_T_ of 6 mL/kg, respiratory rate of 80 breaths/min, inspiratory to expiratory ratio of 1:2, F_I_O_2_ of 21%, and PEEP of 5 cmH_2_O.

Subsequently, a laparotomy was performed, and the animals were euthanized by exsanguination through sections of the abdominal aorta. The trachea was clamped at end-inspiration, and the lungs were removed for further analysis.

### Lung histology

Following lung removal, the right caudal lobes were fixed in 10% buffered formaldehyde. After being embedded in paraffin, 4 µm-thick slices were cut and stained with hematoxylin‒eosin. Morphological tests were performed using a light microscope (Zeiss, Hallbergmoos, Germany) by a pathologist who was blinded to the experimental details. ALI was assessed using the American Thoracic Society-recommended ALI score.^13^ Briefly, 20 random fields filled with pulmonary alveoli were evaluated at 400× magnification and scored individually. Values of 0, 1, or 2 were used to represent injury severity based on the following findings: neutrophils in the alveolar space, neutrophils in the interstitial space, hyaline membranes, proteinaceous debris filling the airspaces, and alveolar septal thickening. To calculate the ALI scores, the sum of the five variables was weighted according to the relevance assigned to each one. The resulting score ranged from 0 (normal) to 1 (most severe injury).

Perivascular edema was quantified through morphometric analysis using the point-counting technique, as previously described.^16^ ^,^ ^17^ At 400× magnification, we analyzed 10 randomly selected fields per lung, focusing on the transversely sectioned intraparenchymatous arteries and veins. The number of points falling within areas of perivascular edema (PE) and those within areas of vessels (V) were computed. The perivascular edema index was calculated as the PE/V ratio. The final perivascular index was the mean of the 10 analyzed fields. All measurements were performed using a blinded method.^16^ ^,^ ^17^

### Clara cell secretory protein-16 (CC16), intercellular adhesion molecule 1 (ICAM-1), and amphiregulin expression

The right cranial lobes were crushed and transferred to microcentrifuge flex tubes containing TRIzol^®^ reagent for total RNA extraction using a standard procedure. cDNA synthesis was carried out using a two-step cDNA synthesis kit (Promega, USA). One microgram of RNA was reverse-transcribed into cDNA via GoScript^TM^ reverse transcriptase (Promega, USA), following the manufacturer’s instructions, resulting in a total reaction volume of 20 μL. Real-time quantitative polynucleotide chain reaction (RT-qPCR) was performed with 5 μL of SYBR Green concentrate in a final volume of 10 μL containing 50 ng of cDNA. To determine the initial relative quantity of cDNA, samples were amplified with ICAM-1, CC16, and amphiregulin primers. The reactions were run on an Applied Biosystems 7500 RT-qPCR machine (Applied Biosystems, USA). An internal standard was established to normalize the relative gene expression level, and this standard was run with each experiment. Melt curve analyses were conducted for all genes, and the specificity and integrity of the PCR products were confirmed by the presence of a single peak. Relative expression was calculated based on the differences in cycle time of the internal standard (GAPDH) compared to that of the target mRNA. Duplicate CT values were analyzed in Microsoft Excel (Microsoft) using the comparative CT method (Applied Biosystems, USA).^14^

### Bronchoalveolar lavage (BAL)

BAL collection was performed in the left lung by instillation and slow aspiration of 4 mL of phosphate-buffered saline solution containing ethylenediamine tetraacetic acid (10 nM). This procedure was repeated three times. Total leucocyte counts were measured in a Neubauer chamber under light microscopy after the samples were diluted in Turk solution. The cell pellet was resuspended in phosphate-buffered saline and stained with May-Grunwald-Giemsa for differential cell counts, which were performed on 300 cells.

### Statistical analysis

Data were expressed as medians and interquartile ranges. The normality of the data was analyzed using the Shapiro-Wilk test. One-way ANOVA was used to compare normally distributed data among groups. To identify differences between the means in the post hoc analysis, Tukey’s pairwise multiple comparison test was applied when a significant *F* ratio was obtained for a factor or for interactions between factors. For non-normally distributed data, the Kruskal-Wallis test was used, followed by the Mann-Whitney U test. Adjustments for multiple comparisons were made using Bonferroni’s correction. All tests were two-tailed, and a p-value < 0.05 was considered statistically significant. The statistical analyses were performed using SPSS software, version 17.0.

## RESULTS

A total of 32 rats were used to form the four groups. During the experimental period, six rats died: two from the C-C group, one from the C-HV group, one from the LPS-C group, and two from the LPS-HV group. In addition, one rat from the C-HV group was excluded because hyperventilation was not achieved by the end of the experiment (Figure 1).

### Hyperventilation induction

NH_4_Cl infusion induced metabolic acidosis, as evidenced by significantly lower levels of HCO_3_^-^ in the C-HV and LPS-HV groups at the end of the experiment compared to baseline values (p < 0.05 for both comparisons). This metabolic acidosis resulted in hyperventilation in these groups, as indicated by the reduction in PvCO_2_ levels after NH_4_Cl infusion compared to baseline values (p < 0.05 for both comparisons) (Table 1).

**Table 1:** Venous blood gas parameters at baseline and at the end of the experiment.

	Group C-C (N = 6)	Group C-HV (N = 6)	Group LPS-C (N = 7)	Group LPS-HV (N = 6)
PvCO_2_ (mmHg) Baseline Final DPvCO_2_	46 (41-47) 57 (40-58) +12 (-7 +19)	43 (42-48) 29 (28-30)* ^#^ -14 (-19 -14)	30 (28-30)*^&^ 30 (27-33)* +2 (-3 +3)	35 (32-38) 26 (26-30)* ^#^ -8 (-9 -7)
pH Baseline Final	7.35 (7.33-7.38) 7.28 (7.19-7.33)	7.38 (7.37-7.38) 7.33 (7.32-7.33)	7.42 (7.37-7.42) 7.38 (7.36-7.38)	7.32 (7.32-7.33)^†^ 7.22 (7.16-7.25)^†^
HCO_3_^-^ (mEq/L) Baseline Final	23 (23-24) 21 (18-22)	25 (25-28) 15 (14-15)* ^#^	18 (16-20)^&^ 17 (15-18)	19 (14-19)^&^ 14 (11-14)*^†#^

C-C: control-control group; C-HV: control-hyperventilation group; LPS-C: lipopolysaccharide-control group; LPS-HV: lipopolysaccharide-hyperventilation group. HCO_3_^-^: bicarbonate; PvCO_2_: venous carbon dioxide partial pressure; D: difference between final and baseline values. Data expressed as median and interquartile range (25% and 75%). * p < 0.05 when compared to C-C; ^&^ p < 0.05 when compared to C-HV; ^†^ p < 0.05 when compared to LPS-C; ^#^ final compared to baseline. Comparisons by Tukey’s pairwise multiple comparison test, with Bonferroni correction.

### Lung histology

**Figure 2:**
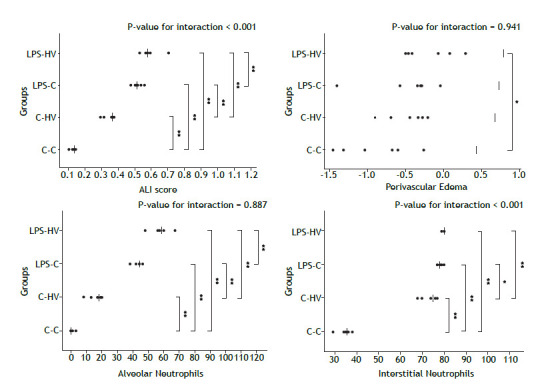
Acute lung injury score, perivascular edema, alveolar neutrophil counts, and interstitial neutrophil counts. ALI: acute lung injury; C‒C: control‒control group; C‒HV: control‒hyperventilation group; LPS‒C: lipopolysaccharide‒control group; LPS‒HV: lipopolysaccharide‒hyperventilation group. Each symbol represents an individual animal, and the black lines represent the median values of each group. * p < 0.05 - Comparisons by Tukey’s pairwise multiple comparison test, with Bonferroni correction.

**Figure 3:**
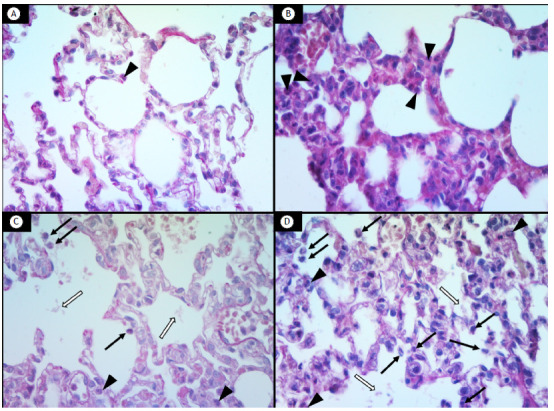
Representative images of histological analysis of the lower right lobe sagittal section stained with hematoxylin‒eosin (H&E, 400×). (A) Control‒control group; (B) Control‒hyperventilation group; (C) LPS‒control group; (D) LPS‒hyperventilation group. Alveolar neutrophils (black arrows); interstitial neutrophils (arrowheads); proteinaceous debris (open arrows). Note that the LPS-hyperventilation group showed higher accumulation of neutrophils in the alveoli when compared to the other groups.

Compared to the animals in the C-C group, the ALI scores of the rats in the LPS-C and LPS-HV groups were significantly higher (p < 0.05 for both comparisons). Analysis of each component of the score revealed that, compared to the C-C group, the LPS-C and LPS-HV groups exhibited greater neutrophil infiltration in both the alveolar and interstitial spaces, as well as increased amounts of alveolar proteinaceous debris (p < 0.05 for all comparisons). Among the rats that received LPS, the ALI score was greater in the LPS-HV group (p < 0.05). This difference was attributed to the greater alveolar and interstitial neutrophil infiltration observed in the LPS-HV group compared to the LPS-C group (p < 0.05 for both comparisons). In comparison with the C-C group, the C-HV group presented a higher ALI score (p < 0.05), with greater alveolar and interstitial neutrophil infiltration (p < 0.05 for both comparisons) (Table 2, Figure 2). Morphometric analysis revealed greater perivascular edema in the LPS-HV group compared to the C-C group (p < 0.05) (Figure 2). Representative histological images of the sagittal section of the right lower lobe, stained with hematoxylin-eosin, are shown in Figure 3.

**Table 2:** Acute lung injury score and its components.

	Groups			
	C-C (N = 6)	C-HV (N = 6)	LPS-C (N = 7)	LPS-HV (N = 6)
Overall score	0.14 (0.12-0.14)	0.36 (0.31-0.37)*	0.51 (0.50-0.54)*^Δ^	0.58 (0.56-0.62)*^Δ†^
Alveolar neutrophils	0.0 (0.0-1.0)	18.0 (11.8-19.3)*	44.0 (42.0-46.0)*^Δ^	58.5 (54.0-61.8)*^Δ†^
Interstitial neutrophils	35.0 (32.8-38.0)	75.0 (69.0-76.3)*	78.0 (77.0-79.0)*^Δ^	80.0 (79.0-80.0)*^Δ^
Proteinaceous debris	2.0 (0.0-4.5)	5.5 (2.8-7.0)	10.0 (8.0-30.0)*	7.5 (3.5-19.0)
Septal thickening	0.0 (0.0-0.5)	4.0 (0.0-5.5)	2.0 (0.0-2.0)	0.5 (0.0-6.3)

C-C: control-control group; C-HV: control-hyperventilation group; LPS-C: lipopolysaccharide-control group; LPS-HV: lipopolysaccharide-hyperventilation group. Data expressed as median and interquartile range (25% and 75%). * p < 0.05 when compared to C-C; Δ p < 0.05 when compared to C-HV; † p < 0.05 when compared to LPS-C. Comparisons by Tukey’s pairwise multiple comparison test, with Bonferroni correction.

### Bronchoalveolar lavage

**Figure 4:**
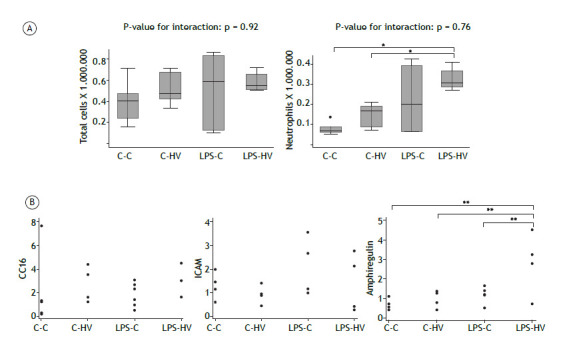
(A) Cell count in the bronchoalveolar lavage (BAL); (B) Relative mRNA levels of Clara cell secretory protein-16 (CC16), intercellular adhesion molecule 1 (ICAM-1), and amphiregulin in lung tissue. GAPDH was used as an internal standard for normalization. C‒C: control‒control group; C‒HV: control‒hyperventilation group; LPS‒C: lipopolysaccharide‒control group; LPS‒HV: lipopolysaccharide‒hyperventilation group. * p < 0.05 - Comparisons by Tukey’s pairwise multiple comparison test, with Bonferroni’s correction. ** p < 0.05 - Comparisons by the Mann-Whitney U test, with Bonferroni correction.

Intraperitoneal LPS injection followed by hyperventilation increased BAL cellularity, as evidenced by the higher number of neutrophils in the LPS-HV group compared to the C-C and C-HV groups (p < 0.05 for both comparisons) (Figure 4).

### ICAM-1, CC16, and amphiregulin mRNA expression

The relative mRNA levels of biological markers associated with inflammation (ICAM-1), pulmonary stretch (amphiregulin), and epithelial cell damage (CC-16) are presented in Figure 4. The relative mRNA expression of amphiregulin was significantly greater in the LPS-HV group compared to the other groups (p < 0.05 for all comparisons) (Figure 4).

## DISCUSSION

In our study, intraperitoneal injection of LPS led to inflammatory ALI, as evidenced by increased ALI scores, with greater alveolar and interstitial neutrophil infiltration in these animals than in the controls. The addition of hyperventilation induced by NH_4_Cl infusion further exacerbated lung injury, as indicated by increased ALI scores, increased perivascular edema, a higher number of neutrophils in the BAL, and elevated mRNA expression of amphiregulin.

Intraperitoneal injection of LPS is an established experimental model of ALI characterized by histological changes (interstitial thickening, alveolar and interstitial neutrophil infiltration, and the presence of proteinaceous debris in the alveolar spaces), altered lung mechanics (increased elastance of the respiratory system), and effects on the alveolar-capillary barrier (increased lung wet/dry weight ratio and elevated albumin concentration in the BAL).^14^ Typically, LPS-induced ALI results in mild impairments in gas exchange, including hypoxemia levels that can be managed without MV.^18^ This characteristic played a decisive role in our selection of the injury model, aligning with our aim to investigate the role of P-SILI during spontaneous, unassisted breathing.

To induce hyperventilation, we applied a metabolic acidosis model through intravenous infusion of NH_4_Cl. This compound is converted into H^+^ and urea in the liver, thus reducing renal HCO_3_^-^ reabsorption. As described by Iwabushi et al. (2003), this model induces metabolic acidosis and compensatory hyperventilation during the infusion period.^19^ Confirming the model’s efficacy, the rats that received NH_4_Cl exhibited lower levels of PvCO_2_ at the end of the experiments compared to baseline values.

We demonstrated that hyperventilation intensified the degree of inflammatory lung injury induced by intraperitoneal LPS injection, as indicated by increased neutrophil infiltration in alveolar spaces observed in the histological analysis and higher neutrophil counts in the BAL. Our findings also revealed elevated expression of amphiregulin, a member of the epidermal growth factor family, which is upregulated in conditions of lung epithelial cell damage, as seen in VILI.^20^ Previous experimental studies have shown that vigorous inspiratory efforts during mechanically-assisted breathing can cause or worsen lung injury. Yoshida et al. (2012) demonstrated in an experimental model of ALI induced by repeated lung lavage that vigorous spontaneous efforts can lead to lung injury, even when the plateau pressure is limited to < 30 cmH_2_O.^6^ Such efforts increase transpulmonary pressure, the total alveolar stretching pressure, resulting in a greater tidal volume. Both elevated transpulmonary pressure and tidal volume are associated with VILI and may be further exacerbated by high respiratory frequency.^6^ Additionally, in heterogeneous lungs, such as in acute respiratory distress syndrome, spontaneous efforts affect the lung regions differently, with greater pleural pressure fluctuations in dependent regions compared to non-dependent ones. This phenomenon, known as *pendelluft*, leads to increased local transpulmonary pressure and tidal inflation in dependent regions, potentially raising the risk of VILI.^7^

Spontaneous efforts also elevate transmural vascular pressure by generating more negative pleural pressure, thereby contributing to pulmonary edema.^21^ Supporting this theory, we demonstrated that rats with inflammatory ALI, where hyperventilation was induced by NH_4_Cl, exhibited a higher perivascular edema index compared to the C-C group.

In contrast with previous studies, our model evaluated the impact of spontaneous efforts on ALI in extubated animals, an area where knowledge remains limited. Positive pressure ventilation can alter the effects of spontaneous efforts on the lungs in multiple ways. For instance, the application of positive end-expiratory pressure reduces *pendelluft* and promotes neuromechanical uncoupling of the diaphragm, both of which mitigate injury.^22^ Furthermore, positive pressure ventilation also influences the transvascular pressure dynamics, adding further complexity to its effects. However, we are not the first to investigate the impact of spontaneous breathing in extubated subjects. Mascheroni et al. (1988) reported that severe hyperventilation during spontaneous breathing led to pulmonary dysfunction in an experimental ovine model with initially healthy lungs.^23^ We built on their findings by examining the impact of hyperventilation on both healthy lungs and lungs previously injured by LPS injection through a factorial design. This approach allowed for a more accurate representation of clinical scenarios in which lung injury from spontaneous efforts may occur, and it helped clarify whether the harmful effects of spontaneous efforts vary based on the underlying lung condition. Our findings indicate that allowing vigorous spontaneous efforts can induce inflammatory injury in previously healthy lungs and exacerbate preexisting inflammatory lung injury.

Our results cannot be directly extrapolated to clinical practice. However, these findings align with data from experimental studies in large animals (all conducted during MV) and with the physiological basis for lung injury caused by spontaneous efforts. Nonetheless, the extent of this injury and its clinical significance in ALI patients remain uncertain. During the COVID-19 pandemic, some authors suggested using noninvasive support (e.g., high-flow nasal oxygen, CPAP, or Bi-PAP) to limit excessive inspiratory efforts in mild cases of acute respiratory failure.^24^ The same authors recommended early intubation, followed by effective sedation, for more severe cases or for patients whose respiratory patterns did not improve with noninvasive support.^11^ The rationale behind these recommendations was the risk of progressive lung function decline (a VILI vortex) driven by patients’ inspiratory efforts.^11^ ^,^ ^24^ Other investigators did not adopt these recommendations, as no clinical study has yet demonstrated that vigorous inspiratory efforts cause clinically significant lung injury.^12^ Moreover, if inspiratory effort-related lung injury exists in this context, clinicians must weigh its risks against those associated with invasive MV to make informed decisions.

The main limitation of our study was the inability to definitively exclude a potential direct effect of NH_4_Cl or metabolic acidosis on lung injury. However, existing experimental evidence suggests that both NH_4_Cl and metabolic acidosis may, in fact, confer a protective effect against VILI by mitigating pulmonary inflammation and reducing pulmonary edema formation, a major manifestation of VILI.^25^ ^-^ ^27^ It is well established that the hypoventilation associated with hypercapnic acidosis is not only well tolerated by individuals with acute lung injury but can also exert protective effects against tissue damage.^28^ Recent experimental studies indicate that metabolic acidosis may also offer protection against acute lung injury. In an *ex vivo* model of acute lung injury induced by ischemia-reperfusion, Laffey et al. (2000) reported that metabolic acidosis, achieved via hydrochloric acid administration, led to reduced lung injury, as evidenced by decreased pulmonary capillary permeability and a lower wet/dry weight ratio.^25^ Additionally, in a VILI model using an isolated ventilated-perfused rabbit lung, the authors showed that metabolic acidosis was as effective as hypercapnic respiratory acidosis in preventing pulmonary edema formation, suggesting that acidosis itself exerts a protective effect against experimental VILI.^26^ Similar protective effects have been reported for NH4Cl-induced metabolic acidosis by Hudalla et al. (2019), who demonstrated that it attenuated lung inflammation in an experimental model of pulmonary hypertension induced by Sugen 5416 and hypoxia. This resulted in reduced lung mRNA levels of proinflammatory mediators (such as tumor necrosis factor, interleukin-6, and C-C motif chemokine ligand 2), as well as decreased plasma interleukin-6 levels.^27^ Considering these findings, along with evidence linking lung injury to excessive inspiratory efforts that overstretch the lung parenchyma, our experimental model of patient self-inflicted lung injury (P-SILI) appears plausible.

Other limitations of this study included the following: 1) The intraperitoneal LPS injection-induced ALI model resulted in metabolic acidosis. Consequently, even before NH_4_Cl infusion, the two groups that received LPS already exhibited acidosis and hyperventilation. Although NH_4_Cl infusion intensified acidosis and hyperventilation in the LPS-HV group, this effect was less pronounced than in the LPS-C group. This smaller difference between the two groups may have limited the detection of hyperventilation-induced injury in LPS-injured lungs. 2) Our model used small animals (Wistar rats), in which regional differences in pleural pressure may not be as significant as in larger animals and humans. Since these regional differences in pleural pressure may play an important role in lung injury from inspiratory efforts, our model might have underestimated the negative impact of hyperventilation in previously injured lungs. 3) The mRNA expression of biological markers was not measured in all animals, resulting in a smaller sample size and reduced statistical power to identify group differences.

In conclusion, our findings demonstrate that hyperventilation exacerbates inflammatory injury in rats with ALI during spontaneous ventilation. These results indicate the need for further investigation into the role of vigorous spontaneous efforts in worsening inflammatory lung injury.
